# Living kidney donation in Brazil, 2010–2023: a descriptive time-series analysis

**DOI:** 10.1590/2175-8239-JBN-2026-0051en

**Published:** 2026-08-21

**Authors:** Márcia Bastos Convento, Fernanda Teixeira Borges

**Affiliations:** 1 Universidade Federal de São Paulo, Escola Paulista de Medicina, Departamento de Medicina, Disciplina de Nefrologia, São Paulo, SP, Brazil.; 2 Universidade Cruzeiro do Sul, Programa de Pós-Graduação Interdisciplinar em Ciências da Saúde, São Paulo, SP, Brazil.

**Keywords:** Kidney Transplantation, Living Donors, Graft Survival, Hospital Mortality

## Abstract

**Introduction:**

Chronic kidney disease imposes a high clinical and economic burden on the Brazilian Unified Health System, and kidney transplantation represents one of the best therapeutic choices for selected patients.

**Objective:**

To describe trends in living-donor (LD) kidney transplantation in Brazil (2010–2023), analyzing the donor–recipient relationship and the operational stock-to-annual production ratio on the waiting list, as well as to compare hospital indicators and estimated patient and graft survival between LD and deceased-donor (DD) kidney transplants.

**Methods:**

This descriptive time-series study used aggregated, publicly available data.

**Results:**

The waiting list increased by 15% (from 33,253 to 38,258), and the total number of transplants rose by 29% (from 4,656 to 6,047). Data showed an increase in deceased-donor transplants (from 3,001 to 5,189) and a decrease in LD transplants (from 1,655 to 858), with the LD share declining from 35.55% to 14.19% and the per-million-population rate falling from 8.8 to 4.2. Among LD, there was a relative decrease in related donors (from 82.80% to 71.21%), a relative increase in unrelated spouse donors (from 10.57% to 18.65%), and a relative increase in other unrelated donors (from 6.64% to 10.14%). In descriptive comparisons, LD transplantation showed higher published patient and graft survival estimates and more favorable hospital indicators, including lower in-hospital mortality, shorter length of stay, and lower mean hospital admission authorization values.

**Conclusion:**

The findings suggest the relevance of integrated strategies to sustain deceased-donor procurement and strengthen living-donor kidney transplantation. However, comparisons between donor types were descriptive and should not be interpreted as causal.

## INTRODUCTION

Kidney diseases affect more than 844 million individuals worldwide and are major contributors to morbidity and premature mortality^1^. Chronic kidney disease (CKD) represents a substantial public health challenge in Brazil and globally^2^. An estimated 6.7% of the Brazilian adult population is affected by CKD, a proportion that nearly triples among individuals aged 60 years or older. Between 2010 and 2023, hospitalizations coded as CKD increased from 84,337 to 140,648, while CKD-related mortality rose from 2.9 to 4.0 deaths per 100,000 inhabitants^2^.

Over the same period, spending by the Brazilian Unified Health System (SUS) on CKD care increased markedly. Outpatient expenditures on hemodialysis and peritoneal dialysis exceeded R$ 38.3 billion between 2010 and 2023, representing an increase of approximately 73% over the analyzed period^2^. Hemodialysis with up to three sessions per week accounted for the largest share of this expenditure, surpassing R$ 27.5 billion^2^. Hospital expenditures also rose substantially, reaching a cumulative total of R$ 3.18 billion between 2010 and 2023^2^.

This pattern is consistent with growing demand and increasing clinical complexity in the care of patients with CKD in Brazil. Population aging, combined with the high prevalence of hypertension, diabetes mellitus, and cardiovascular disease, likely contributes to the consolidation of CKD as one of the leading public health challenges in the contemporary global context^3^. Improvements in care have been associated with longer survival among patients receiving dialysis and with expanded treatment coverage. At the same time, the incorporation of new technologies and shifts in clinical practice require greater planning and resource allocation to effectively organize nephrology care^4^.

In this context, kidney transplantation stands out as a therapeutic modality capable of restoring renal function in patients with advanced CKD and, compared with dialysis, is associated with longer life expectancy, lower morbidity, and better quality of life^5,6^. Among transplant modalities, living-donor (LD) kidney transplantation offers additional advantages over deceased-donor (DD) transplantation, including higher graft survival rates and, in many cases, shorter time to transplantation, making it the preferred option whenever feasible^7,8^. From an organizational perspective, LD donation may help reduce pressure on the waiting list and improve procedural predictability, thereby potentially optimizing resource use and care pathways^9,10^, provided that donor risks are rigorously assessed and monitored.

Despite the well-established advantages of living-donor kidney transplantation, its long-term evolution in Brazil remains insufficiently characterized at the national level. Although the decline in living-donor transplantation is recognized by transplant professionals, analyses specifically focused on its temporal trajectory and on changes in donor–recipient relationship patterns remain limited. A plausible explanation is that this decline may reflect the interaction of multiple contextual factors, including demographic and epidemiological changes, socioeconomic barriers, donor-protection mechanisms, health-system organization, and regulatory aspects. These factors may affect both the availability of medically eligible living donors and access to this transplantation modality.

Therefore, this study aimed to describe trends in kidney transplantation in Brazil from 2010 to 2023, with emphasis on temporal patterns in living-donor transplantation, donor–recipient relationship patterns, waiting-list indicators, and descriptive comparisons of clinical indicators, including patient and graft survival, as well as hospital-related indicators between living-donor and deceased-donor transplantation.

## METHODS

This descriptive time-series study adopted a quantitative approach and analyzed aggregated, publicly available secondary data from Brazil covering the period from 2010 to 2023. The series was truncated in 2023 because the 2024 report did not provide a detailed breakdown of the LD profile by donor–recipient relationship. Data sources included the Hospital Information System of the Brazilian Unified Health System (SIH/SUS), accessed via DATASUS^11^, the National Transplant System^12^ (SNT), and the Brazilian Transplant Registry of the Brazilian Association of Organ Transplantation^13^ (RBT/ABTO). No individual-level linkage across databases was performed; therefore, comparisons across sources were descriptive in nature and may reflect differences in coverage, operational definitions, and indicator consolidation methods.

From SIH/SUS^11^ (DATASUS), aggregated data were extracted using TABNET by procedure and place of hospitalization for the period 2010–2023 and stratified by macroregion (North, Northeast, Southeast, South, and Central-West). Kidney transplant hospitalizations were identified using the SIGTAP codes 0505020092 (kidney transplant from a deceased donor) and 0505020106 (kidney transplant from a living donor) as the main procedure. For each year and macroregion, we obtained the mean hospital length of stay (days), the in-hospital mortality rate (%), and the mean Hospital Admission Authorization (AIH, R$) value for hospitalizations in which kidney transplantation was recorded as the main procedure.

To summarize indicators for the 2010–2023 period, AIH-weighted annual means were calculated to reflect yearly procedure volume; as a sensitivity analysis, simple arithmetic means of annual estimates (with equal weight assigned to each year) were also computed. Monetary values correspond to nominal amounts recorded in the AIH and were not adjusted for inflation; therefore, they do not represent real changes in costs over time. As SIH/SUS is an administrative database, changes in procedure and payment tables, billing rules, and recording practices over time may affect comparability across years and macroregions. SIH/SUS captures hospitalizations financed by the SUS based on AIH issuance and processing by accredited public and private facilities.

In the SNT^12^, the transplant waiting list corresponds to the Technical Registry of active and semi-active potential recipients, as defined in official reports. For this analysis, we used annual totals from the historical kidney transplant waiting list series, which served as the primary metric. The “general waiting list” reported by the SNT was used only for contextual purposes, as applicable, and its composition may vary over time (e.g., inclusion of different organs or tissues in certain reporting periods). These data represent a stock of records in the Technical Registry and do not capture the dynamics of entries to and exits from the waiting list throughout the year.

From the RBT/ABTO^13^, we obtained annual totals of kidney transplants by donor type (living and deceased), the distribution of donor–recipient relationships among LD donations, transplant rates per million population (pmp), and estimates of patient and graft survival following kidney transplantation. The pmp rates were extracted directly from published RBT/ABTO reports, without recalculation. Survival estimates were derived from the RBT/ABTO Survival Registry, initiated in January 2010. They were analyzed at the published follow-up time points (1, 2, 3, 5, 7, 10, and 14 years), following the definitions of follow-up, censoring, losses, and graft failure described in the registry documentation, including the estimation methods reported by the RBT/ABTO.

To characterize the relationship between waiting-list stock and annual transplant production, we combined, by calendar year, the stock of patients registered in the SNT with the yearly number of kidney transplants performed, as reported by the RBT/ABTO. Waiting-list stock was denoted as E, and the annual number of kidney transplants performed was denoted as T. We then calculated the operational difference between waiting-list stock and annual transplant production as E − T, and the operational stock–annual production indicator as (E − T)/E, equivalent to 1 − (T/E). This approach allowed the construction of operational stock-to-production indicators (including the absolute difference between registry stock and annual transplant volume and derived proportions). These indicators were intended solely for descriptive purposes and were not interpreted as individual probabilities of transplantation, waiting times, or direct measures of unmet demand.

Variables were analyzed descriptively using absolute and relative frequencies and, when applicable, transplant rates per million population (pmp), yearly percentage variation (YV%), overall percentage change between 2010 and 2023, and compound annual growth rate (CAGR). Yearly percentage variation was calculated as [(current-year value − prior-year value) / prior-year value] × 100. Overall percentage change was calculated as [(2023 value − 2010 value) / 2010 value] × 100. The CAGR was calculated as [(final value / initial value)^(1/n) − 1] × 100, where n represents the number of annual intervals; for 2010–2023, n = 13. CAGR was calculated using absolute transplant counts for deceased-donor and living-donor kidney transplants. No hypothesis testing was performed, and all results are presented exclusively in descriptive form.

Because this study was based on aggregated, publicly accessible secondary data and did not involve identifiable individual-level information, submission to a Research Ethics Committee was not required, in accordance with National Health Council Resolution No. 510/2016.

## RESULTS

### Kidney transplant waiting list: stock, composition, and the stock–flow relationship

**Figure 1:**
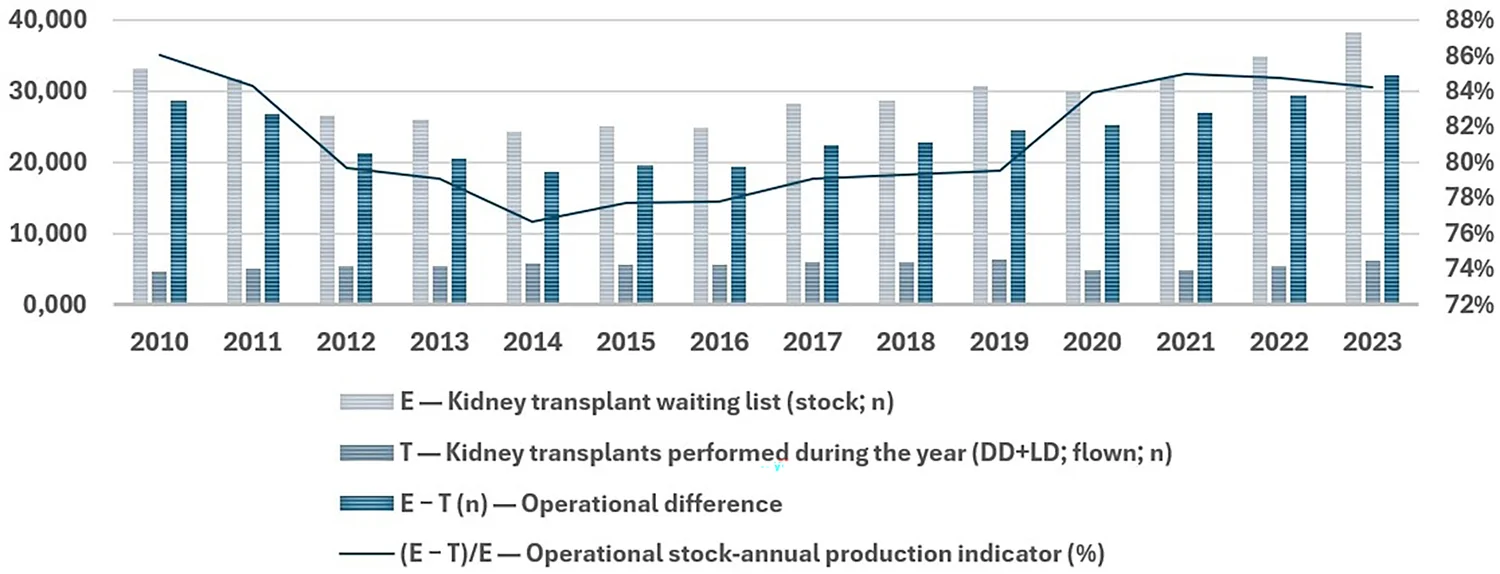
Trends in the waiting-list stock (E) of patients listed for kidney transplantation (Technical Registry: active and semi-active; SNT) and the annual number of kidney transplants performed (T) (living and deceased donors; RBT/ABTO), Brazil, 2010–2023.

Figure 1 illustrates trends in the waiting-list stock (E) of patients listed for kidney transplantation in Brazil (Technical Registry: active and semi-active) and the annual number of kidney transplants performed (T), including both living- and deceased-donor procedures, between 2010 and 2023. Over the study period, the waiting-list stock increased from 33,253 to 38,258 patients (+15.0%), whereas the annual number of transplants performed rose from 4,656 to 6,047 (+29.9%).

Bars represent E (n), T (n), and the operational difference E − T (n). The line shows the operational stock–annual production indicator (E − T) / E = 1 − (T/E), in % (right axis; scale expanded for visualization). This is a comparison between the annual recorded stock and the annual transplant production, and it should not be interpreted as an individual probability of transplantation, waiting time, unmet demand, or the “proportion of the list served” in the year.

Data sources: SNT^12^ and RBT/ABTO^13^.

Over the same interval, the operational difference (E − T) increased from 28,597 to 32,211 (+12.6%). This metric was defined as the difference between the total number of patients on the waiting list reported by the SNT and the number of kidney transplants performed during the same year, according to the Brazilian Transplant Registry. The operational stock–annual production indicator, expressed as the proportion of the waiting-list stock exceeding the yearly transplant volume, ranged from 77% to 86% across the series. This indicator was 86% in 2010, declined gradually through 2014 (77%), remained relatively stable between 2015 and 2019 (78%–80%), and increased again from 2020 onward (84% in 2020, 85% in 2021 and 2022, and 84% in 2023).

### Kidney transplant activity by donor type in Brazil, 2010–2023

Table 1 presents trends in kidney transplant activity in Brazil between 2010 and 2023, including total transplant rates per million population (pmp), donor-specific rates, absolute numbers of deceased-donor (DD) and living-donor (LD) transplants, and annual percentage change. Over the study period, the total kidney transplant rate increased from 23.3 to 29.8 pmp. During the same period, the number of DD transplants increased from 3,001 to 5,189 (+72.9%), whereas the number of LD transplants declined from 1,655 to 858 (−48.2%), indicating opposing temporal trajectories between donor types. The compound annual growth rates were 4.30% for DD and −4.93% for LD.

**Table 1:** Kidney transplant activity in Brazil, 2010–2023: rates per million population (PMP), number of deceased-donor (DD) and living-donor (LD) transplants, and percent yearly variation (YV%).

Year	DD (pmp)	LD (pmp)	Total (pmp)	DD (n)	DD (YV%)	LD (n)	LD (YV%)
2010	14.5	8.8	23.3	3,001	–	1,655	–
2011	16.2	8.2	24.4	3,325	10.80	1,650	–0.30
2012	17.3	7.2	24.5	3,914	17.71	1,499	–9.15
2013	21.4	7.3	28.6	4,065	3.86	1,382	–7.81
2014	22.4	7.3	29.7	4,278	5.40	1,388	0.43
2015	21.7	5.9	27.6	4,403	2.92	1,190	–14.27
2016	21.1	6.0	27.1	4,311	–2.09	1,220	2.52
2017	23.3	5.5	28.8	4,796	11.25	1,140	–6.56
2018	23.7	4.9	28.7	4,927	2.73	1,025	–10.09
2019	25.0	5.2	30.2	5,225	6.05	1,078	5.17
2020	20.8	2.1	22.9	4,380	–16.17	446	–58.63
2021	19.7	2.7	22.4	4,186	–4.43	593	32.96
2022	21.4	3.4	24.9	4,579	9.39	738	24.45
2023	25.6	4.2	29.8	5,189	13.32	858	16.26

Note − Percent yearly variation (YV%) was calculated relative to the preceding year. Overall change between 2010 and 2023 was calculated as [(2023 value − 2010 value) / 2010 value] × 100. Over the study period, deceased-donor (DD) kidney transplants increased by 72.90%, whereas living-donor (LD) kidney transplants decreased by 48.16%. The CAGR, calculated using 13 annual intervals and absolute transplant counts, was +4.30% per year for DD and −4.93% per year for LD. Rates per million population (pmp) were used as reported in the RBT/ABTO reports.

Data source: RBT/ABTO^13^

Between 2010 and 2015, DD transplants increased steadily, reaching 4,403 procedures, while LD transplants declined to 1,190. In 2016, DD transplants showed a slight decrease (4,311), whereas LD transplants exhibited a one-time increase (1,220). From 2017 to 2019, DD transplants resumed growth, reaching 5,225 procedures, while LD transplants continued an overall downward trend, with minor fluctuations, totaling 1,078 procedures in 2019. In parallel, the DD transplant rate increased from 14.5 pmp in 2010 to 25.0 pmp in 2019, whereas the LD transplant rate declined from 8.8 to 5.2 pmp.

In 2020, transplant activity declined sharply in the context of the COVID-19 pandemic. DD transplants fell to 4,380 (−16.17% compared with 2019), and LD transplants dropped to 446 (−58.63%). The total kidney transplant rate declined to 22.9 pmp in 2020 and 22.4 pmp in 2021. During the same period, the DD transplant rate decreased to 20.8 pmp in 2020 and 19.7 pmp in 2021, whereas the LD transplant rate fell more markedly, reaching 2.1 pmp in 2020 and 2.7 pmp in 2021.

From 2022 onward, transplant activity recovered for both donor types. In 2023, DD transplants reached 5,189, the highest value observed in the series, corresponding to 25.6 pmp. LD transplants increased to 858 in 2023, with a rate of 4.2 pmp, but remained below the levels observed at the beginning of the series. Based on the counts shown in Table 1, the share of LD transplants among all kidney transplants decreased from 35.55% in 2010 to 14.19% in 2023, indicating a growing predominance of DD kidney transplantation in national transplant activity. Over the study period, DD kidney transplants increased by 72.90%, whereas LD kidney transplants decreased by 48.16%. The compound annual growth rate (CAGR), calculated using 13 annual intervals and absolute transplant counts, was +4.30% per year for DD and −4.93% per year for LD.

### Relative share of kidney transplants performed with DD and LD

Table 2 presents the relative share of kidney transplants performed with DD and LD in Brazil between 2010 and 2023. Over the study period, the proportion of transplants performed with DD increased from 64.45% to 85.81%, whereas the share of LD decreased from 35.55% to 14.19%. Consequently, the difference between the two modalities (DD − LD) widened from 28.90 to 71.62 percentage points (p.p.).

**Table 2:** Relative share of kidney transplants from deceased donors (DD) and living donors (LD), and the absolute difference between shares (DD–LD), in percentage points (p.p.), Brazil, 2010–2023.

Year	DD (%)	LD (%)	Difference (DD–LD) p.p.
2010	64.45	35.55	28.90
2011	66.83	33.17	33.66
2012	72.31	27.69	44.62
2013	74.63	25.37	49.26
2014	75.50	24.50	51.00
2015	78.72	21.28	57.44
2016	77.94	22.06	55.88
2017	80.79	19.21	61.58
2018	82.78	17.22	65.56
2019	82.90	17.10	65.80
2020	90.76	9.24	81.52
2021	87.59	12.41	75.18
2022	86.12	13.88	72.24
2023	85.81	14.19	71.62

Note – Percentages were calculated as DD / (DD + LD) × 100; %LD = 100−%DD; difference expressed in percentage points (p.p.).

Data source: RBT/ABTO^13^

Up to 2015, this difference increased progressively, from 28.90 p.p. in 2010 to 57.44 p.p. in 2015. Between 2016 and 2019, the difference remained high, ranging from 55.88 to 65.80 p.p. In 2020, the difference reached its highest value in the series (81.52 p.p.), coinciding with a sharp decline in the LD share of total kidney transplants during the COVID-19 pandemic. Between 2021 and 2023, the difference narrowed, reaching 71.62 p.p. in 2023, although it remained above levels observed before 2020.

### Profile of living donors by donor–recipient relationship

Table 3 presents trends in LD kidney transplants in Brazil from 2010 to 2023, showing a reduction in overall volume accompanied by changes in the donor–recipient relationship profile. The total number of LD transplants declined from 1,655 in 2010 to 858 in 2023, representing an absolute reduction of 797 procedures and a relative decrease of 48.2%.

**Table 3:** Absolute number (n) and share (%) of living-donor kidney transplants in Brazil, by donor–recipient relationship (related; unrelated-spouse; unrelated-others), 2010–2023.

Year	Related, n (%)	Unrelated spouse, n (%)	Unrelated others, n (%)	Total LD, n
2010	1,370 (82.80)	175 (10.57)	110 (6.64)	1,655
2011	1,352 (81.94)	185 (11.21)	113 (6.85)	1,650
2012	1,226 (81.79)	196 (13.08)	77 (5.14)	1,499
2013	1,129 (81.69)	186 (13.46)	67 (4.85)	1,382
2014	1,105 (79.61)	187 (13.48)	96 (6.92)	1,388
2015	965 (81.09)	166 (13.95)	59 (4.96)	1,190
2016	963 (78.93)	173 (14.18)	84 (6.89)	1,220
2017	899 (78.86)	158 (13.86)	83 (7.28)	1,140
2018	815 (79.51)	148 (14.44)	62 (6.05)	1,025
2019	833 (77.27)	165 (15.31)	80 (7.42)	1,078
2020	320 (71.75)	82 (18.39)	44 (9.86)	446
2021	434 (73.20)	97 (16.35)	62 (10.45)	593
2022	535 (72.50)	121 (16.40)	82 (11.11)	738
2023	611 (71.21)	160 (18.65)	87 (10.14)	858

Note − Percentages were calculated relative to the annual total number of living-donor transplants.

Data source: RBT/ABTO^13^

Related donors remained the predominant source of living donation throughout the period. However, their absolute number decreased from 1,370 to 611 procedures, and their proportional share declined from 82.80% to 71.21%. Between 2010 and 2016, related-donor volumes remained relatively high, ranging from 1,370 to 963 procedures, with proportional shares between 78.93% and 82.80%. From 2017 onward, the absolute number declined more clearly, reaching the lowest value in 2020 (320 procedures), followed by partial recovery to 434 in 2021, 535 in 2022, and 611 in 2023. Despite this recovery in volume, the proportional share remained below 74% after 2020 and reached the lowest value in the series in 2023.

Among unrelated spouse donors, the absolute number changed from 175 in 2010 to 160 in 2023, with a marked decline in 2020 followed by recovery in subsequent years. In proportional terms, however, the share increased from 10.57% to 18.65%. Among unrelated donors classified as “others,” the absolute number declined from 110 to 87, with the lowest value also observed in 2020, followed by gradual recovery; proportionally, this category increased from 6.64% to 10.14%, exceeding 10% from 2021 onward.

**Figure 2:**
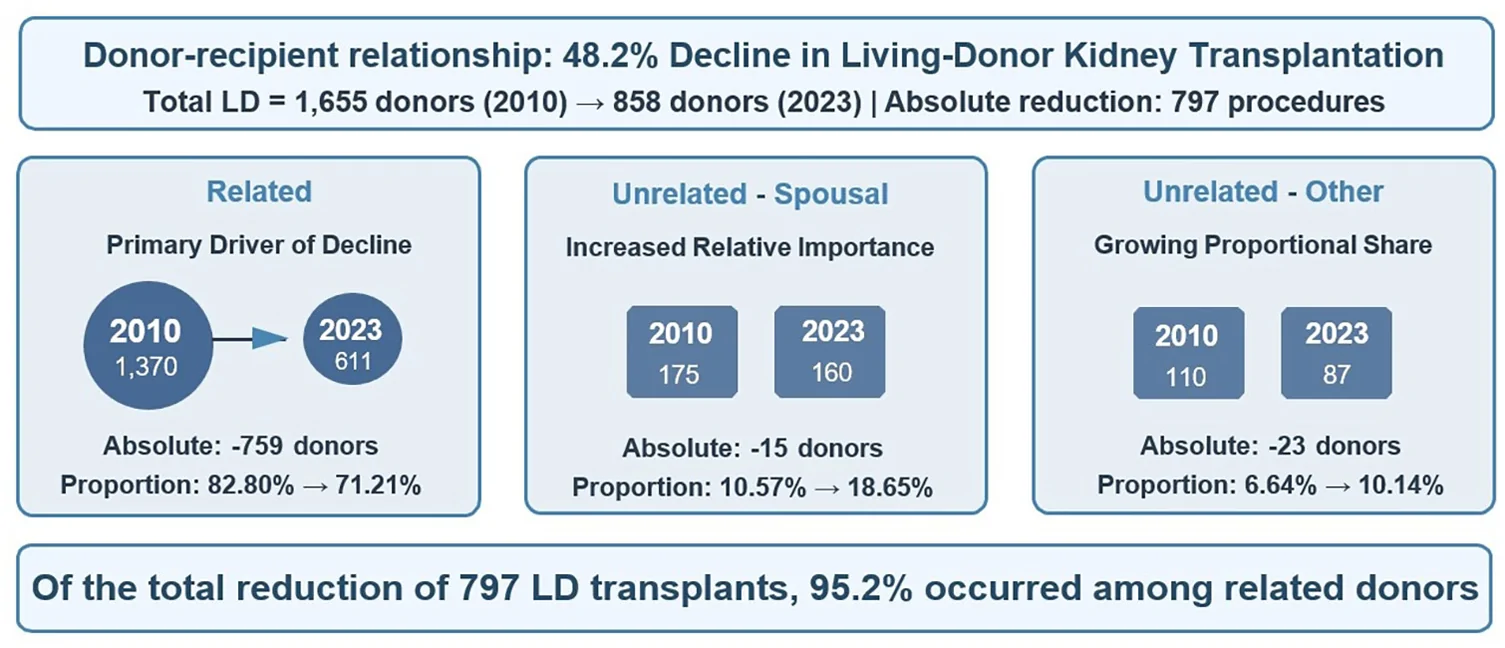
Comparison of living-donor (LD) kidney transplants in Brazil between 2010 and 2023, by donor–recipient relationship: related, unrelated-spouse, and unrelated-other donors, with absolute (n) and proportional (%) changes.

Overall, the decline in LD transplantation reflected both a reduction in volume and a shift in donor–recipient composition. Figure 2 illustrates the 48.2% decline in living-donor kidney transplant procedures between 2010 and 2023, from 1,655 to 858 donors, according to donor–recipient relationship. The decline was driven predominantly by related donors, who accounted for 759 of the 797 fewer procedures, corresponding to 95.2% of the total reduction. Unrelated-spousal and unrelated-other donors also decreased in absolute numbers, although their combined relative share increased from 17.2% to 28.8%. Related and unrelated-spousal donors together accounted for 774 fewer procedures, or 97.1% of the overall reduction.

Data source: RBT/ABTO^13^.

### Estimated patient and graft survival after kidney transplantation, by donor type

Table 4 presents estimates of patient and graft survival after kidney transplantation, as reported by the RBT/ABTO, by donor type and at follow-up time points (1, 2, 3, 5, 7, 10, and 14 years). At all evaluated time points, patient survival was higher in the LD group than in the DD group: 97% versus 92% at 1 year (a difference of 5 percentage points); 93% versus 83% at 5 years (10 percentage points); and 83% versus 67% at 14 years (16 percentage points). Graft survival was also consistently higher among LD recipients: 94% versus 86% at 1 year (8 percentage points); 85% versus 70% at 5 years (15 percentage points); and 65% versus 44% at 14 years (21 percentage points). For both patient and graft survival, the differences between donor types widened with increasing follow-up duration.

**Table 4:** Patient and graft survival after kidney transplantation, by donor type (living-donor and deceased-donor), at 1, 2, 3, 5, 7, 10, and 14 years of follow-up (values in %).

	Survivel curve					
	Patient			Graft		
Year	LD (%)	DD (%)	Difference patient (p.p.)	LD (%)	DD (%)	Difference graft (p.p.)
1^st^	97	92	5	94	86	8
2^nd^	97	90	7	92	82	10
3^rd^	95	88	7	90	78	12
5^th^	93	83	10	85	70	15
7^th^	91	79	12	80	63	17
10^th^	87	73	14	72	53	19
14^th^	83	67	16	65	44	21

Note – Differences between groups are presented in percentage points (p.p.), calculated as LD − DD. Estimates follow the definitions of follow-up, censoring/loss, and graft-failure criteria described in the RBT/ABTO and are presented descriptively in this analysis.

Data source: RBT/ABTO^13^

### Hospital indicators by donor type

Table 5 compares hospital indicators for SUS-financed kidney transplant hospitalizations by donor type and macroregion from 2010 to 2023. The indicators include mean length of stay, in-hospital mortality, and mean AIH values. For DD transplants, the mean length of stay ranged from 11.8 days in the Southeast to 13.9 days in the Central-West, with a national mean of 12.3 days. For LD transplants, the mean length of stay was consistently lower across all macroregions, ranging from 9.2 days in the Southeast to 11.3 days in the Northeast, with a national mean of 9.9 days. This corresponds to a relative difference of −19.51% compared with the national mean for DD transplants.

**Table 5:** Mean hospital length of stay, in-hospital mortality (%), and mean recorded/reimbursed AIH values for sus-financed kidney transplant hospitalizations, by donor type (DD and LD) and macroregions, Brazil, 2010–2023.

Deceased-donor (DD)	Mean length of stay (days)	In-hospital mortality (%)	Mean AIH value (R$, nominal)
North	13.20	1.41	36,418.25
Northeast	12.10	1.18	41,218.74
Southeast	11.80	2.00	41,578.19
South	12.90	1.48	43,469.54
Central-West	13.90	1.93	38,945.81
Brazil	12.30	1.70	41,717.97
**Living donor (LD)**	**Mean length of stay (days)**	**In-hospital mortality (%)**	**Mean AIH value (R$, nominal)**
North	9.60	0.40	26,051.61
Northeast	11.30	0.45	25,637.28
Southeast	9.20	0.40	29,662.84
South	11.00	0.57	28,159.50
Central-West	9.80	0.64	27,493.66
Brazil	9.90	0.45	28,595.01
**Difference (LD vs. DD), Brazil (%)**	**Mean length of stay (days)**	**In-hospital mortality (%)**	**Mean AIH value (R$, nominal)**
	–19.51%	–73.53%	–31.46%

Note – The relative difference (LD vs. DD) was calculated as [(LD − DD) / DD] × 100, based on national period means. Monetary values represent the mean amount recorded/reimbursed in the AIH in current (nominal) Brazilian reais, without inflation adjustment, and do not correspond to total economic cost. Values were extracted from the DATASUS/SIH-SUS database and are presented as originally reported in the source system.

Data source: SIH/SUS – Brazilian ministry of health^11^

In-hospital mortality for DD transplants ranged from 1.18% in the Northeast to 2.00% in the Southeast, with a national mean of 1.70%. For LD transplants, in-hospital mortality ranged from 0.40% in the North and Southeast to 0.64% in the Central-West, with a national mean of 0.45%, representing a relative difference of −73.53% compared with the national mean for DD transplants. Regarding the mean AIH values recorded and reimbursed, DD transplants ranged from R$ 36,418.25 in the North to R$ 43,469.54 in the South, with a national mean of R$ 41,717.97. For LD transplants, mean AIH values were lower across all macroregions, ranging from R$ 25,637.28 in the Northeast to R$ 29,662.84 in the Southeast, with a national mean of R$ 28,595.01. This corresponds to a relative difference of −31.46% for LD compared with the national mean for DD transplants.

## DISCUSSION

Between 2010 and 2023, the overall kidney transplant rate increased from 23.3 to 29.8 pmp, while the operational difference between the recorded kidney waiting-list stock and annual transplant production varied slightly, from 86% to 84%. During the same period, deceased-donor transplant rates increased from 14.5 to 25.6 pmp, whereas living-donor rates declined from 8.8 to 4.2 pmp. Consequently, the donor-source profile changed markedly, with the living-donor share decreasing from 35.55% to 14.19% and the deceased-donor share increasing from 64.45% to 85.81%.

This national profile differs from the global distribution reported by the Global Observatory on Donation and Transplantation, in which approximately 62% of kidney transplants are performed with deceased donors and 38% with living donors^14^. This contrast suggests the need to examine contextual factors that may be associated with the lower relative participation of LD transplantation in Brazil. Several non-mutually exclusive factors may contribute to this pattern.

At the population level, aging and the high prevalence of overweight (59.7%), obesity (24.6%), diabetes (10.2%), prediabetes (12%–14%), and hypertension (26.8%, exceeding 50% among older adults) may be associated with a smaller pool of medically eligible potential living kidney donors, particularly within family donor networks, where related candidates may share familial, environmental, and socioeconomic patterns, as well as behavioral factors linked to renal and cardiometabolic risk^15,16,17,18^.

Contemporary living-donor evaluation extends beyond excluding clinically manifest disease and incorporates the estimation of future risk. Thus, previously undiagnosed, underestimated, or insufficiently monitored risk factors may only emerge during the transplant-evaluation pathway, making donor selection more time-consuming, resource-intensive, and emotionally burdensome, particularly when the candidate is ultimately not approved for donation. This should be interpreted as a plausible contextual hypothesis rather than as direct explanatory evidence.

International comparisons are informative in this regard. Türkiye, Israel, and the Republic of Korea, three of the four highest-ranked countries for living-donor kidney transplantation, illustrate how cultural, economic, legal, and organizational contexts may be associated with different living-donation patterns^14^. In Türkiye, the predominance of living donation should be interpreted within a context of donor protection and health-system organization, including labor regulations that may help preserve employment during recovery, sickness benefits during recovery, and public coverage of donor evaluation, surgery, hospitalization, and post-donation follow-up within a nationally organized transplant system^19^.

In Israel, transplant legislation and donor-support mechanisms reportedly include cost compensation, reimbursement of lost wages, temporary exemption from health-related fees, state coverage for life and disability insurance, and future priority on the transplant waiting list. The country is also notable for a high proportion of living kidney donations from unrelated donors, described in association with altruistic donation and organized support for living donors^20^.

In the Republic of Korea, most living kidney donors are family members, and living-donor kidney transplantation has been examined in relation to cultural and demographic factors, including Confucian-influenced perceptions of bodily integrity after death, demographic changes, declining rates of some major causes of brain death, population aging, and family-centered values such as responsibility and mutual obligation^21^.

These examples are pertinent to Brazil because barriers may persist even when direct medical care is publicly covered. Although the SUS covers donor evaluation, surgery, hospitalization, and follow-up, indirect barriers remain, including the absence of specific job-stability protection for living kidney donors and medical leave mechanisms that may primarily benefit formally employed workers.

Although medical leave mechanisms may primarily benefit formally employed workers, informal workers may remain particularly vulnerable. PNAD *Contínua* data for 2023, the final year of the study period, showed 39.4 million informal workers and an informality rate of 39.2%^22^. Thus, time away from work, transportation costs, caregiving responsibilities, and income insecurity may represent socioeconomic and logistical barriers for potential donors.

In parallel, the Brazilian series also showed changes in the donor–recipient relationship profile among LD kidney transplants. Related donors remained the predominant source of living donation throughout the series; however, their proportional share declined from 82.80% in 2010 to 71.21% in 2023. In contrast, the proportional share of spouse donors increased from 10.57% to 18.65%, and that of other unrelated donors increased from 6.64% to 10.14%, although absolute numbers declined in both unrelated categories.

This point is relevant to the present findings because the decline in living-donor kidney transplantation was concentrated among related and unrelated-spouse donors, groups for whom judicial authorization is not required^23^; together, they accounted for 97.1% of the total reduction over the study period. This finding suggests that educational, organizational, and donor-protection strategies should be considered alongside regulatory debate.

The changing composition of living donation is clinically relevant because, in the present descriptive analysis, published RBT/ABTO survival estimates for the same period analyzed in this study, 2010–2023, showed that, at up to 14 years of follow-up, LD recipients had 16- and 21-percentage-point higher patient and graft survival, respectively, than DD recipients^13^. However, these registry-based estimates are descriptive and may reflect differences in donor and recipient selection, clinical profiles, time on dialysis, immunological compatibility, ischemia time, and procedural logistics.

Hospital indicators also showed a favorable descriptive profile for LD transplantation. In the SIH/SUS database^11^, LD-associated hospitalizations had shorter length of stay (−19.51%), lower in-hospital mortality (−73.53%), and lower mean AIH reimbursement values (−31.46%) than DD-associated hospitalizations across all macroregions. This profile may reflect the logistical predictability and elective nature of many LD procedures, without implying causality; AIH values represent nominal recorded/reimbursed amounts and may not capture total economic costs, particularly those related to donor evaluation, nephrectomy, follow-up, and potential complications.

Although descriptive, these clinical and hospital indicators suggest the relevance of identifying barriers to LD transplantation that may be addressed through health-system interventions. In this context, educational interventions represent one approach to addressing potentially modifiable barriers to LD transplantation^24,25^. In the Renal Education and Choices at Home (ReACH) study^25^, a home-based program delivered by specialist nurses to patients, family members, and friends aimed to increase knowledge, promote dialogue about donation, and reduce barriers to transplantation. After the intervention, 53% of patients initiated evaluation of at least one potential donor.

Consistent with these findings, in a qualitative review of 27 international studies, Truhan et al.^26^ identified the late or fragmented provision of information about LD donation as a recurrent barrier, associated with uncertainty, overestimation of donor risk, and difficulties in family communication. The authors emphasized the importance of early, continuous, family-centered, and professionally mediated education, with involvement of the family network from the early stages^26,27^.

Beyond educational strategies, kidney paired donation has been proposed as a strategy to increase LD transplantation opportunities in highly structured systems; however, Medina-Pestana et al.^28^ describe barriers to its broad implementation in Latin American and Brazilian contexts, including socioeconomic inequalities, access asymmetries, and ethical, logistical, and equity-related challenges. Nevertheless, recent initiatives, including Bill No. 3,903/2024, indicate ongoing national regulatory debate on paired donation^29^.

Based on the descriptive findings presented, it is reasonable to consider integrated educational, organizational, and regulatory actions to support living kidney donation and improve transplant-access pathways, alongside continued strengthening of deceased-donor procurement and strategies to reduce pressure on the kidney transplant waiting list, which is the largest organ-specific transplant waiting list in Brazil. In this context, awareness initiatives and ethically appropriate donor-support measures should be considered in decision-making by the SUS and health insurance companies.

### Study limitations

This study has limitations inherent to the use of secondary, aggregated, and administrative data, which restrict analyses to the population level and prevent examination of individual determinants of living donation (e.g., age, sex, education, family relationship, and clinical conditions). The use of different information systems (SNT, RBT/ABTO, and SIH/SUS) may lead to divergences in operational definitions, annual closing procedures, completeness, and record consistency, potentially resulting in underreporting, variability across regions and institutions, and information bias. SNT waiting-list measures describe stock in the Technical Registry and do not capture the dynamics of entries and exits over time. In addition, comparisons between donation modalities are descriptive and unadjusted for differences in clinical profile, care complexity (case mix), elective status, and logistical factors. Finally, SIH/SUS indicators reflect hospitalizations within the SUS, with mortality restricted to the hospitalization period, and the mean AIH value represents the recorded/reimbursed amount in nominal terms rather than total economic cost; administrative, coding, and reimbursement changes over the period may affect comparability.

## CONCLUSION

Between 2010 and 2023, kidney transplantation in Brazil showed a marked shift in donor profile, characterized by an increase in deceased-donor procedures and a reduction in living-donor procedures. During the same period, the kidney transplant waiting-list stock remained substantially higher than annual transplant production, suggesting a persistent operational burden on the transplant system. Among living-donor transplants, the data also indicated changes in donor–recipient relationship patterns, with a more pronounced decline among related donors and an increased relative participation of unrelated donors, particularly unrelated spouse donors. The clinical and hospital indicators were descriptively more favorable among living-donor kidney transplant recipients, including higher patient and graft survival, shorter length of stay, lower in-hospital mortality, and lower mean AIH values. Taken together, these findings suggest the relevance of integrated strategies to strengthen living-donor kidney transplantation alongside continued efforts to sustain deceased-donor procurement. However, this study is descriptive and should not be interpreted as causal.

## Data Availability

The datasets analyzed during the current study were derived from publicly available sources cited in the manuscript.
