# Supplementary material for: Living kidney donation in Brazil, 2010–2023: a descriptive time-series analysis
Source: J Bras Nefrol. 2026 Aug 21;48(4):e20260051. doi: 10.1590/2175-8239-JBN-2026-0051en (PMC13544939; doi:10.1590/2175-8239-JBN-2026-0051en)
Supplement: Translation

# Translation: Doação renal em vida no Brasil, 2010–2023: uma análise descritiva de série temporal

**DOI:** 10.1590/2175-8239-JBN-2026-0051pt

**Authors:** Márcia Bastos Convento, Fernanda Teixeira Borges

**Affiliations:** 1 Universidade Federal de São Paulo, Escola Paulista de Medicina, Departamento de Medicina, Disciplina de Nefrologia, São Paulo, SP, Brasil.; 2 Universidade Cruzeiro do Sul, Programa de Pós-Graduação Interdisciplinar em Ciências da Saúde, São Paulo, SP, Brasil.

**Keywords:** Transplante Renal, Doadores Vivos, Sobrevida do Enxerto, Mortalidade Hospitalar

## Abstract

**Introdução:**

A doença renal crônica representa uma expressiva carga clínica e econômica para o Sistema Único de Saúde brasileiro, e o transplante renal configura-se como uma das melhores alternativas terapêuticas para pacientes criteriosamente selecionados.

**Objetivo:**

Descrever as tendências do transplante renal com doador vivo no Brasil, entre 2010 e 2023, considerando vínculo doador–receptor, indicadores da lista de espera, indicadores hospitalares e estimativas de sobrevida do paciente e do enxerto, em comparação aos transplantes com doador falecido.

**Métodos:**

Trata-se de um estudo descritivo de série temporal, baseado em dados agregados, de domínio público.

**Resultados:**

A lista de espera aumentou 15%, de 33.253 para 38.258 pacientes, enquanto o número total de transplantes cresceu 29%, de 4.656 para 6.047 procedimentos. Esse aumento foi impulsionado pelos transplantes com doador falecido, que passaram de 3.001 para 5.189, enquanto os transplantes com doador vivo diminuíram de 1.655 para 858. A participação dos transplantes intervivos caiu de 35,55% para 14,19%, e a taxa pmp reduziu de 8,8 para 4,2. Entre os doadores vivos, houve redução proporcional da categoria Parente, de 82,80% para 71,21%, e aumento relativo das categorias Não Parente: Cônjuge, de 10,57% para 18,65%, e Não Parente: Outros, de 6,64% para 10,14%. Nas comparações descritivas, o transplante com doador vivo apresentou estimativas superiores de sobrevida do paciente e do enxerto, além de indicadores hospitalares mais favoráveis.

**Conclusão:**

Os achados ressaltam a importância de estratégias integradas capazes de sustentar a captação de órgãos de doadores falecidos e, simultaneamente, fortalecer o transplante renal intervivos no Brasil. Contudo, as comparações entre os tipos de doador têm caráter descritivo e não devem ser interpretadas como evidência de relação causal.

## INTRODUÇÃO

As doenças renais afetam mais de 844 milhões de indivíduos em todo o mundo e constituem importantes determinantes de morbidade e mortalidade prematura^1^. A doença renal crônica (DRC) representa um expressivo desafio de saúde pública, tanto no Brasil quanto no cenário global^2^. Estima-se que 6,7% da população adulta brasileira seja acometida por DRC, proporção que praticamente triplica entre indivíduos com 60 anos ou mais. Entre 2010 e 2023, as internações codificadas como DRC aumentaram de 84.337 para 140.648, enquanto a mortalidade relacionada à DRC aumentou de 2,9 para 4,0 óbitos por 100.000 habitantes^2^.

No mesmo período, os gastos do Sistema Único de Saúde (SUS) com a DRC aumentaram de forma expressiva. As despesas ambulatoriais com hemodiálise e diálise peritoneal ultrapassaram R$ 38,3 bilhões entre 2010 e 2023, representando um aumento de aproximadamente 73% ao longo do período analisado^2^. A hemodiálise com até três sessões semanais respondeu pela maior parcela desse dispêndio, superando R$ 27,5 bilhões^2^. As despesas hospitalares também aumentaram substancialmente, alcançando um total acumulado de R$ 3,18 bilhões entre 2010 e 2023^2^.

A expansão dos gastos ambulatoriais e hospitalares observada no período acompanha a ampliação da demanda assistencial e a maior complexidade clínica do cuidado prestado a pacientes com DRC no Brasil. Nesse cenário, o envelhecimento populacional, aliado à elevada prevalência de hipertensão arterial, diabetes mellitus e doenças cardiovasculares, contribui para consolidar a DRC como um dos principais desafios de saúde pública no contexto global contemporâneo^3^. No entanto, avanços assistenciais têm sido associados à maior sobrevida de pacientes em diálise e à ampliação da cobertura terapêutica. Ao mesmo tempo, a incorporação de novas tecnologias e as mudanças na prática clínica exigem maior planejamento e melhor alocação de recursos para a organização efetiva da atenção nefrológica^4^.

Nesse contexto, o transplante renal se destaca como uma modalidade terapêutica capaz de restabelecer a função renal em pacientes com DRC avançada e, quando comparado à diálise, está associado a uma maior expectativa de vida, menor morbidade e melhor qualidade de vida^5,6^. Entre as modalidades de transplante, o transplante renal com doador vivo (DV) oferece vantagens adicionais em relação ao transplante com doador falecido (DF), incluindo maiores taxas de sobrevida do enxerto e, em muitos casos, menor tempo até o transplante, configurando-se como a opção preferencial sempre que factível^7,8^. Sob a perspectiva organizacional, a doação intervivos pode contribuir para reduzir a pressão sobre a lista de espera e aumentar a previsibilidade do procedimento, favorecendo potencialmente a otimização do uso de recursos e dos fluxos assistenciais^9,10^, desde que os riscos ao doador sejam rigorosamente avaliados e monitorados.

Apesar das vantagens bem estabelecidas do transplante renal com doador vivo, sua trajetória temporal no Brasil permanece pouco explorada em estudos de abrangência nacional. Embora o declínio do transplante intervivos seja reconhecido por profissionais da área de transplantes, ainda são limitadas as análises voltadas especificamente à sua trajetória temporal e às alterações na distribuição dos vínculos entre doadores e receptores. Uma explicação plausível é que esse declínio reflita a interação de múltiplos fatores contextuais, incluindo mudanças demográficas e epidemiológicas, barreiras socioeconômicas, mecanismos de proteção ao doador, organização do sistema de saúde e aspectos regulatórios. Esses fatores podem repercutir tanto na disponibilidade de potenciais doadores vivos quanto na possibilidade de os pacientes se beneficiarem do transplante renal com doador vivo.

Portanto, este estudo teve como objetivo descrever as tendências do transplante renal no Brasil entre 2010 e 2023, com ênfase nos padrões temporais do transplante com doador vivo, nas categorias de vínculo doador–receptor, nos indicadores da lista de espera e nas comparações descritivas de indicadores clínicos, incluindo a sobrevida do paciente e do enxerto, bem como de indicadores hospitalares entre transplantes com doador vivo e com doador falecido.

## MÉTODOS

Este estudo descritivo de série temporal adotou abordagem quantitativa e analisou dados secundários agregados, de acesso público, referentes ao Brasil, no período de 2010 a 2023. A série foi encerrada em 2023 porque o relatório de 2024 não apresentou o detalhamento do perfil dos doadores vivos segundo o vínculo doador–receptor. As fontes de dados compreenderam o Sistema de Informações Hospitalares do Sistema Único de Saúde (SIH/SUS), acessado por meio do DATASUS^11^, o Sistema Nacional de Transplantes (SNT)^12^ e o Registro Brasileiro de Transplantes da Associação Brasileira de Transplante de Órgãos (RBT/ABTO)^13^. Não foi realizado pareamento em nível individual entre as bases de dados; assim, as comparações entre as fontes tiveram caráter descritivo e podem refletir diferenças de cobertura, definições operacionais e métodos de consolidação dos indicadores.

A partir do SIH/SUS^11^ (DATASUS), foram extraídos dados agregados por meio do TABNET, segundo procedimento e local de internação, para o período de 2010 a 2023, estratificados por macrorregião: Norte, Nordeste, Sudeste, Sul e Centro-Oeste. As internações para transplante renal foram identificadas pelos códigos SIGTAP 0505020092, referente ao transplante renal com doador falecido, e 0505020106, referente ao transplante renal com doador vivo, registrados como procedimento principal. Para cada ano e macrorregião, foram obtidos o tempo médio de permanência hospitalar, em dias, a taxa de mortalidade intra-hospitalar, em percentual, e o valor médio da Autorização de Internação Hospitalar (AIH), em reais (R$), moeda nacional, referentes às internações nas quais o transplante renal foi registrado como procedimento principal.

Para sintetizar os indicadores referentes ao período de 2010 a 2023, foram calculadas médias anuais ponderadas pelo número de AIH, a fim de refletir o volume anual de procedimentos. Como análise de sensibilidade, também foram calculadas médias aritméticas simples das estimativas anuais, atribuindo o mesmo peso a cada ano. Os valores monetários correspondem aos montantes nominais registrados nas AIH e não foram ajustados pela inflação; portanto, não representam variações reais dos custos ao longo do tempo. Como o SIH/SUS é uma base administrativa, mudanças nas tabelas de procedimentos e pagamentos, nas regras de faturamento e nas práticas de registro ao longo do tempo podem afetar a comparabilidade entre anos e macrorregiões. O SIH/SUS registra internações financiadas pelo SUS com base na emissão e no processamento das AIH por estabelecimentos públicos e privados credenciados.

No SNT^12^, a lista de espera para transplante corresponde ao Registro Técnico de potenciais receptores ativos e semiativos, conforme definido nos relatórios oficiais. Para esta análise, foram utilizados os totais anuais da série histórica da lista de espera para transplante renal, adotados como métrica principal. A lista geral de espera reportada pelo SNT foi utilizada apenas para fins contextuais, uma vez que sua composição pode variar ao longo do tempo, por exemplo, com a inclusão de diferentes órgãos em determinados períodos de notificação. Esses dados representam um estoque de registros no Registro Técnico e não capturam a dinâmica de entradas e saídas da lista de espera ao longo do ano.

A partir do RBT/ABTO^13^, foram obtidos os totais anuais de transplantes renais segundo o tipo de doador, vivo ou falecido, a distribuição dos vínculos doador–receptor entre as doações intervivos, as taxas de transplante por milhão de população (pmp) e as estimativas de sobrevida do paciente e do enxerto após o transplante renal. As taxas pmp foram extraídas diretamente dos relatórios publicados pelo RBT/ABTO, sem recálculo. As estimativas de sobrevida foram derivadas do Registro de Sobrevida do RBT/ABTO, iniciado em janeiro de 2010. Essas estimativas foram analisadas nos pontos de seguimento publicados, correspondentes a 1, 2, 3, 5, 7, 10 e 14 anos, de acordo com as definições de seguimento, censura, perdas e falência do enxerto descritas na documentação do registro, incluindo os métodos de estimação reportados pelo RBT/ABTO.

Para caracterizar a relação entre o estoque da lista de espera e a produção anual de transplantes, combinamos, por ano-calendário, o estoque de pacientes registrados no SNT com o número anual de transplantes renais realizados, conforme reportado pelo RBT/ABTO. O estoque da lista de espera foi denominado E, e o número anual de transplantes renais realizados foi denominado T. Em seguida, calculamos a diferença operacional entre o estoque da lista de espera e a produção anual de transplantes como E − T, bem como o indicador operacional estoque-produção anual como (E − T) / E, equivalente a 1 − (T / E). Essa abordagem permitiu a construção de indicadores operacionais da relação entre estoque e produção, como a diferença absoluta entre o estoque registrado e o volume anual de transplantes, além das proporções derivadas.

Esses indicadores foram utilizados exclusivamente para fins descritivos e não foram interpretados como probabilidades individuais de transplante, tempos de espera ou medidas diretas de demanda não atendida.

As variáveis foram analisadas de forma descritiva, por meio de frequências absolutas e relativas e, quando aplicável, taxas de pmp, variação percentual anual (VPA%), variação percentual global entre 2010 e 2023 e taxa composta de crescimento anual (CAGR). A variação percentual anual foi calculada como [(valor do ano corrente − valor do ano anterior) / valor do ano anterior] × 100. Já a variação percentual global foi calculada como [(valor de 2023 − valor de 2010) / valor de 2010] × 100. A CAGR foi calculada como [(valor final / valor inicial)^(1/n) − 1] × 100, em que n representa o número de intervalos anuais; para o período de 2010 a 2023, n = 13. Para esse cálculo, foram utilizadas as contagens absolutas de transplantes renais com doador falecido e com doador vivo. Não foram realizados testes de hipóteses, e todos os resultados são apresentados exclusivamente de forma descritiva.

Como este estudo se baseou em dados secundários agregados e de acesso público, sem envolver informações individualmente identificáveis, não foi necessária sua submissão a um Comitê de Ética em Pesquisa, em conformidade com a Resolução nº 510/2016 do Conselho Nacional de Saúde.

## RESULTADOS

### Lista de espera para transplante renal: estoque, composição e relação entre estoque e fluxo

A Figura 1 apresentou as tendências do estoque da lista de espera (E) de pacientes inscritos para transplante renal no Brasil, considerando o Registro Técnico de potenciais receptores ativos e semiativos, e do número anual de transplantes renais realizados (T), incluindo procedimentos com doador vivo e com doador falecido, entre 2010 e 2023. Ao longo do período avaliado, o estoque da lista de espera aumentou de 33.253 para 38.258 pacientes (+15,0%), enquanto o número anual de transplantes realizados cresceu de 4.656 para 6.047 (+29,9%).

**Figura 1:** Tendências do estoque da lista de espera (E) de pacientes inscritos para transplante renal (Registro Técnico: ativos e semiativos; SNT) e do número anual de transplantes renais realizados (T) (doadores vivos e falecidos; RBT/ABTO), Brasil, 2010–2023.

As barras representam E (n), T (n) e a diferença operacional E − T (n). A linha mostra o indicador operacional estoque-produção anual, (E − T) / E = 1 − (T / E), em % (eixo direito; escala ampliada para visualização). Trata-se de uma comparação entre o estoque anual registrado e a produção anual de transplantes e não deve ser interpretada como probabilidade individual de transplante, tempo de espera, demanda não atendida ou “proporção da lista atendida” no ano.

Fontes dos dados: SNT^12^ e RBT/ABTO^13^.

No mesmo intervalo, a diferença operacional (E − T), definida como a subtração entre o número total de pacientes na lista de espera informado pelo SNT e o número de transplantes renais realizados no ano segundo o Registro Brasileiro de Transplantes, aumentou de 28.597 para 32.211 (+12,6%). O indicador operacional estoque-produção anual, expresso como a proporção do estoque da lista de espera que excedia o volume anual de transplantes, variou de 77% a 86% ao longo da série. Esse indicador foi de 86% em 2010, reduziu gradualmente até 2014 (77%) e manteve-se relativamente estável entre 2015 e 2019 (78% a 80%), voltando a aumentar a partir de 2020, atingindo 84% em 2020, 85% em 2021 e 2022, e 84% em 2023.

### Atividade de transplante renal segundo o tipo de doador no Brasil, 2010–2023

Na Tabela 1, foram apresentadas as tendências da atividade de transplante renal no Brasil entre 2010 e 2023, incluindo as taxas totais de pmp, as taxas específicas segundo o tipo de doador, os números absolutos de transplantes com DF e com DV, e a variação percentual anual. Ao longo do período estudado, a taxa total de transplante renal aumentou de 23,3 para 29,8 pmp. No mesmo período, o número de transplantes com DF aumentou de 3.001 para 5.189 (+72,9%), enquanto o número de transplantes com DV diminuiu de 1.655 para 858 (−48,2%), indicando trajetórias temporais opostas entre os tipos de doador. As taxas compostas de crescimento anual foram de 4,30% para DF e de −4,93% para DV.

**Tabela 1:** Atividade de transplante renal no Brasil, 2010–2023: taxas por milhão de população (PMP), número de transplantes com doador falecido (DF) e com doador vivo (DV), e variação percentual anual (VPA%).

Ano	DF (pmp)	DV (pmp)	Total (pmp)	DF (n)	DF (VPA%)	DV (n)	DV (VPA%)
2010	14,5	8,8	23,3	3,001	–	1,655	–
2011	16,2	8,2	24,4	3,325	10,80	1,650	−0,30
2012	17,3	7,2	24,5	3,914	17,71	1,499	−9,15
2013	21,4	7,3	28,6	4,065	3,86	1,382	−7,81
2014	22,4	7,3	29,7	4,278	5,40	1,388	0,43
2015	21,7	5,9	27,6	4,403	2,92	1,190	−14,27
2016	21,1	6,0	27,1	4,311	−2,09	1,220	2,52
2017	23,3	5,5	28,8	4,796	11,25	1,140	−6,56
2018	23,7	4,9	28,7	4,927	2,73	1,025	−10,09
2019	25,0	5,2	30,2	5,225	6,05	1,078	5,17
2020	20,8	2,1	22,9	4,380	−16,17	446	−58,63
2021	19,7	2,7	22,4	4,186	−4,43	593	32,96
2022	21,4	3,4	24,9	4,579	9,39	738	24,45
2023	25,6	4,2	29,8	5,189	13,32	858	16,26

Nota − A variação percentual anual (VPA%) foi calculada em relação ao ano anterior. A variação global entre 2010 e 2023 foi calculada como [(valor de 2023 − valor de 2010) / valor de 2010] × 100. Ao longo do período estudado, os transplantes renais com doador falecido (DF) aumentaram 72,90%, enquanto os transplantes renais com doador vivo (DV) diminuíram 48,16%. A taxa composta de crescimento anual (CAGR), calculada com base em 13 intervalos anuais e nas contagens absolutas de transplantes, foi de +4,30% ao ano para DF e de −4,93% ao ano para DV. As taxas por milhão de população (pmp) foram utilizadas conforme reportadas nos relatórios do RBT/ABTO.

Fonte dos dados: RBT/ABTO^13^

Entre 2010 e 2015, os transplantes com DF aumentaram de forma sustentada, alcançando 4.403 procedimentos, enquanto os transplantes com DV diminuíram para 1.190. Em 2016, os transplantes com DF apresentaram discreta redução (4.311), ao passo que os transplantes com DV tiveram um aumento pontual (1.220). De 2017 a 2019, os transplantes com DF retomaram a trajetória de crescimento, atingindo 5.225 procedimentos, enquanto os transplantes com DV mantiveram tendência geral de queda, com pequenas flutuações, totalizando 1.078 procedimentos em 2019. Paralelamente, a taxa de transplantes com DF aumentou de 14,5 pmp em 2010 para 25,0 pmp em 2019, enquanto a taxa de transplantes com DV diminuiu de 8,8 para 5,2 pmp.

Em 2020, a atividade de transplante apresentou acentuada redução no contexto da pandemia de COVID-19. Os transplantes com DF diminuíram para 4.380, o que correspondeu a uma redução de 16,17% em relação a 2019, e os transplantes com DV caíram para 446, uma redução de 58,63%. A taxa total de transplante renal diminuiu para 22,9 pmp em 2020 e 22,4 pmp em 2021. No mesmo período, a taxa de transplantes com DF reduziu para 20,8 pmp em 2020 e 19,7 pmp em 2021, enquanto a taxa de transplantes com DV apresentou queda mais expressiva, atingindo 2,1 pmp em 2020 e 2,7 pmp em 2021.

A partir de 2022, a atividade de transplante se recuperou em ambos os tipos de doador. Em 2023, os transplantes com DF alcançaram 5.189 procedimentos, o maior valor observado na série, correspondente a 25,6 pmp. Os transplantes com DV aumentaram para 858 em 2023, com taxa de 4,2 pmp, mas permaneceram abaixo dos níveis registrados no início da série. Com base nas contagens apresentadas na Tabela 1, a participação dos transplantes com DV entre todos os transplantes renais diminuiu de 35,55% em 2010 para 14,19% em 2023, indicando crescente predominância do transplante renal com DF na atividade nacional de transplantes. Ao longo do período estudado, os transplantes renais com DF aumentaram 72,90%, enquanto os transplantes renais com DV diminuíram 48,16%.

### Participação relativa dos transplantes renais realizados com DF e DV

A Tabela 2 apresentou a participação relativa dos transplantes renais realizados com DF e DV no Brasil entre 2010 e 2023. Ao longo do período estudado, a proporção de transplantes realizados com DF aumentou de 64,45% para 85,81%, enquanto a participação dos transplantes com DV diminuiu de 35,55% para 14,19%. Consequentemente, a diferença entre as duas modalidades (DF − DV) ampliou-se de 28,90 para 71,62 pontos percentuais (p.p.).

**Tabela 2:** Participação relativa dos transplantes renais com doador falecido (DF) e com doador vivo (DV), e diferença absoluta entre as participações (DF–DV), em pontos percentuais (p.p.), Brasil, 2010–2023.

Ano	DF (%)	DV (%)	Diferença (DF–DV) p.p.
2010	64,45	35,55	28,90
2011	66,83	33,17	33,66
2012	72,31	27,69	44,62
2013	74,63	25,37	49,26
2014	75,50	24,50	51,00
2015	78,72	21,28	57,44
2016	77,94	22,06	55,88
2017	80,79	19,21	61,58
2018	82,78	17,22	65,56
2019	82,90	17,10	65,80
2020	90,76	9,24	81,52
2021	87,59	12,41	75,18
2022	86,12	13,88	72,24
2023	85,81	14,19	71,62

Nota – Os percentuais foram calculados como DF/(DF+DV) × 100; %DV = 100 − %DF; a diferença foi expressa em pontos percentuais (p.p.).

Fonte dos dados: RBT/ABTO^13^

Até 2015, essa diferença aumentou progressivamente, passando de 28,90 p.p. em 2010 para 57,44 p.p. em 2015. Entre 2016 e 2019, a diferença permaneceu elevada, variando de 55,88 a 65,80 p.p. Em 2020, a diferença atingiu o maior valor da série (81,52 p.p.), coincidindo com uma queda acentuada da participação dos transplantes com DV no total de transplantes renais durante a pandemia de COVID-19. Entre 2021 e 2023, a diferença reduziu, alcançando 71,62 p.p. em 2023, embora tenha permanecido acima dos níveis observados antes de 2020.

### Perfil dos doadores vivos segundo o vínculo doador–receptor

A Tabela 3 apresentou as tendências dos transplantes renais com doador vivo no Brasil entre 2010 e 2023, evidenciando redução do volume total acompanhada por mudanças na composição das categorias de vínculo doador–receptor. O número total de transplantes com DV diminuiu de 1.655 em 2010 para 858 em 2023, o que representou uma redução absoluta de 797 procedimentos e uma queda relativa de 48,2%.

**Tabela 3:** Número absoluto (n) e participação (%) dos transplantes renais com doador vivo no Brasil, segundo o vínculo doador–receptor (parente; não parente: cônjuge; não parente: outros), 2010–2023.

Ano	Parente (n, %)	Não parente: cônjuge (n, %)	Não parente: outros (n, %)	Total DV (n)
2010	1,370 (82,80)	175 (10,57)	110 (6,64)	1,655
2011	1,352 (81,94)	185 (11,21)	113 (6,85)	1,650
2012	1,226 (81,79)	196 (13,08)	77 (5,14)	1,499
2013	1,129 (81,69)	186 (13,46)	67 (4,85)	1,382
2014	1,105 (79,61)	187 (13,48)	96 (6,92)	1,388
2015	965 (81,09)	166 (13,95)	59 (4,96)	1,190
2016	963 (78,93)	173 (14,18)	84 (6,89)	1,220
2017	899 (78,86)	158 (13,86)	83 (7,28)	1,140
2018	815 (79,51)	148 (14,44)	62 (6,05)	1,025
2019	833 (77,27)	165 (15,31)	80 (7,42)	1,078
2020	320 (71,75)	82 (18,39)	44 (9,86)	446
2021	434 (73,20)	97 (16,35)	62 (10,45)	593
2022	535 (72,50)	121 (16,40)	82 (11,11)	738
2023	611 (71,21)	160 (18,65)	87 (10,14)	858

Nota − Os percentuais foram calculados em relação ao número total anual de transplantes com doador vivo.

Fonte dos dados: RBT/ABTO^13^

A categoria Parente permaneceu como a principal fonte de doação intervivos ao longo de todo o período. Entretanto, seu número absoluto diminuiu de 1.370 para 611 procedimentos, e sua participação proporcional reduziu de 82,80% para 71,21%. Entre 2010 e 2016, os transplantes na categoria Parente se mantiveram em patamar superior ao observado nos anos subsequentes, variando de 1.370 a 963 procedimentos, com participações proporcionais entre 78,93% e 82,80%. A partir de 2017, foi observado um declínio mais evidente no número absoluto, que atingiu o menor valor em 2020, com 320 procedimentos, seguido de recuperação parcial para 434 em 2021, 535 em 2022 e 611 em 2023. Apesar dessa recuperação em volume, a participação proporcional permaneceu abaixo de 74% após 2020 e atingiu, em 2023, o menor valor da série (71,21%).

Na categoria Não Parente: Cônjuge, o número absoluto passou de 175 em 2010 para 160 em 2023, com declínio acentuado em 2020, seguido de recuperação nos anos subsequentes. Em termos proporcionais, entretanto, a participação aumentou de 10,57% para 18,65%. Na categoria Não Parente: Outros, o número absoluto diminuiu de 110 para 87, com o menor valor também observado em 2020, após o qual ocorreu recuperação gradual; proporcionalmente, essa categoria aumentou de 6,64% para 10,14%, ultrapassando 10% a partir de 2021.

De modo geral, o declínio dos transplantes com DV refletiu tanto a redução global do volume quanto uma mudança na composição proporcional das categorias de vínculo doador–receptor. A Figura 2 ilustrou a redução de 48,2% nos transplantes renais com doador vivo entre 2010 e 2023, de 1.655 para 858 doadores, de acordo com a relação doador–receptor. Essa queda foi predominantemente impulsionada pelos doadores aparentados, que responderam por 759 dos 797 procedimentos a menos, correspondendo a 95,2% da redução total. Os doadores não aparentados cônjuges e os não aparentados outros também apresentaram redução em números absolutos, embora sua participação relativa combinada tenha aumentado de 17,2% para 28,8%. Em conjunto, os doadores aparentados e os não aparentados cônjuges responderam por 774 procedimentos a menos, ou 97,1% da redução global.

**Figura 2:** Comparação dos transplantes renais com doador vivo (DV) no Brasil em 2023 em relação a 2010, segundo o vínculo doador–receptor: Parente, Não Parente: Cônjuge e Não Parente: Outros, com variações absolutas (n) e proporcionais (%).

Fonte dos dados: RBT/ABTO^13^.

### Sobrevida estimada do paciente e do enxerto após o transplante renal, segundo o tipo de doador

A Tabela 4 apresentou as estimativas de sobrevida do paciente e do enxerto após o transplante renal, conforme reportadas pelo RBT/ABTO, segundo o tipo de doador e os pontos de seguimento avaliados: 1, 2, 3, 5, 7, 10 e 14 anos, com início em 2010. Em todos os pontos analisados, a sobrevida do paciente foi maior no grupo DV do que no grupo DF: 97% *versus* 92% em 1 ano, com diferença de 5 pontos percentuais; 93% *versus* 83% em 5 anos, com diferença de 10 pontos percentuais; e 83% *versus* 67% em 14 anos, com diferença de 16 pontos percentuais. A sobrevida do enxerto também foi consistentemente maior entre receptores de DV: 94% *versus* 86% em 1 ano, com diferença de 8 pontos percentuais; 85% *versus* 70% em 5 anos, com diferença de 15 pontos percentuais; e 65% *versus* 44% em 14 anos, com diferença de 21 pontos percentuais. Tanto para a sobrevida do paciente quanto para a sobrevida do enxerto, as diferenças entre os tipos de doador aumentaram com o maior tempo de seguimento.

**Tabela 4:** Sobrevida do paciente e do enxerto após o transplante renal, segundo o tipo de doador, doador vivo (DV) e doador falecido (DF), nos pontos de seguimento de 1, 2, 3, 5, 7, 10 e 14 anos, a partir de 2010, com valores expressos em %.

	Sobrevida					
	Paciente			Enxerto		
Ano	DV (%)	DF (%)	Diferença Paciente (p.p.)	DV (%)	DF (%)	Diferença Enxerto (p.p.)
1º	97	92	5	94	86	8
2º	97	90	7	92	82	10
3º	95	88	7	90	78	12
5º	93	83	10	85	70	15
7º	91	79	12	80	63	17
10º	87	73	14	72	53	19
14º	83	67	16	65	44	21

Nota – As diferenças entre os grupos são apresentadas em pontos percentuais (p.p.), calculadas como DV − DF. As estimativas seguiram as definições de seguimento, censura/perda e critérios de falência do enxerto descritos pelo RBT/ABTO e são apresentadas de forma descritiva nesta análise.

Fonte dos dados: RBT/ABTO^13^

### Indicadores hospitalares segundo o tipo de doador

A Tabela 5 comparou os indicadores hospitalares das internações para transplante renal financiadas pelo SUS, segundo o tipo de doador e a macrorregião, no período de 2010 a 2023. Os indicadores incluíram tempo médio de permanência, mortalidade intra-hospitalar e valores médios de AIH. Para os transplantes com DF, o tempo médio de permanência variou de 11,8 dias no Sudeste a 13,9 dias no Centro-Oeste, com média nacional de 12,3 dias. Para os transplantes com DV, o tempo médio de permanência foi consistentemente menor em todas as macrorregiões, variando de 9,2 dias no Sudeste a 11,3 dias no Nordeste, com média nacional de 9,9 dias. Esse resultado correspondeu a uma diferença relativa de −19,51% em comparação com a média nacional dos transplantes com DF.

**Tabela 5:** Tempo médio de permanência hospitalar, mortalidade intra-hospitalar (%) e valores médios registrados/reembolsados (AIH) das internações para transplante renal financiadas pelo sus, segundo o tipo de doador (DF e DV) e macrorregiões, Brasil, 2010–2023.

Doador falecido (DF)	Permanência hospitalar (dias)	Mortalidade intra-hospitalar (%)	Valores médios da AIH (R$, nominais)
Norte	13,20	1,41	36.418,25
Nordeste	12,10	1,18	41.218,74
Sudeste	11,80	2,00	41.578,19
Sul	12,90	1,48	43.469,54
Centro-Oeste	13,90	1,93	38.945,81
Brasil	12,30	1,70	41.717,97
**Doador vivo (DV)**	**Permanência hospitalar (dias)**	**Mortalidade intra-hospitalar (%)**	**Valores médios da AIH (R$, nominais)**
Norte	9,60	0,40	26.051,61
Nordeste	11,30	0,45	25.637,28
Sudeste	9,20	0,40	29.662,84
Sul	11,00	0,57	28.159,50
Centro-Oeste	9,80	0,64	27.493,66
Brasil	9,90	0,45	28.595,01
**Diferença (DV vs. DF), Brasil (%)**	**Permanência hospitalar (dias)**	**Mortalidade intra-hospitalar (%)**	**Valores médios da AIH (R$, nominais)**
	–19,51%	–73,53%	–31,46%

Nota – A diferença relativa (DV em relação a DF) foi calculada como [(DV − DF) / DF] × 100, com base nas médias nacionais do período. Os valores monetários representaram o valor médio registrado/reembolsado na AIH, em reais correntes, nominais, sem ajuste pela inflação, e não correspondem ao custo econômico total. Os valores foram extraídos da base DATASUS/SIH-SUS e são apresentados conforme originalmente reportados no sistema de origem.

Fonte dos dados: SIH/SUS – (DATASUS)^11^

A mortalidade intra-hospitalar nos transplantes com DF variou de 1,18% no Nordeste a 2,00% no Sudeste, com média nacional de 1,70%. Nos transplantes com DV, a mortalidade intra-hospitalar variou de 0,40% no Norte e no Sudeste a 0,64% no Centro-Oeste, com média nacional de 0,45%, representando uma diferença relativa de −73,53% em comparação com a média nacional dos transplantes com DF. Quanto aos valores médios de AIH registrados e reembolsados, os transplantes com DF variaram de R$ 36.418,25 no Norte a R$ 43.469,54 no Sul, com média nacional de R$ 41.717,97. Para os transplantes com DV, os valores médios de AIH foram menores em todas as macrorregiões, variando de R$ 25.637,28 no Nordeste a R$ 29.662,84 no Sudeste, com média nacional de R$ 28.595,01. Esse resultado correspondeu a uma diferença relativa de −31,46% para os transplantes com DV em comparação com a média nacional dos transplantes com DF.

## DISCUSSÃO

Entre 2010 e 2023, a taxa global de transplante renal aumentou de 23,3 para 29,8 pmp, enquanto o indicador operacional da relação entre o estoque registrado da lista de espera para transplante renal e a produção anual de transplantes variou discretamente, de 86% para 84%. No mesmo período, as taxas de transplante com doador falecido aumentaram de 14,5 para 25,6 pmp, ao passo que as taxas de transplante com doador vivo diminuíram de 8,8 para 4,2 pmp. Consequentemente, o perfil da fonte doadora se modificou de forma expressiva, com redução da participação dos transplantes com doador vivo de 35,55% para 14,19% e aumento da participação dos transplantes com doador falecido de 64,45% para 85,81%.

Esse perfil observado no Brasil contrasta com a distribuição global descrita pelo *Global Observatory on Donation and Transplantation*, na qual aproximadamente 62% dos transplantes renais são realizados com doadores falecidos e 38% com doadores vivos^14^. Essa divergência sugere a necessidade de examinar fatores contextuais potencialmente associados à menor participação relativa do transplante com DV no Brasil. Diversos fatores, não mutuamente excludentes, podem contribuir para esse cenário.

Em nível populacional, o envelhecimento e a elevada prevalência na população adulta de excesso de peso (59,7%), obesidade (24,6%), diabetes (10,2%), pré-diabetes (12%–14%) e hipertensão arterial (26,8%, ultrapassando 50% entre idosos) podem estar associados a um contingente menor de potenciais doadores renais vivos clinicamente elegíveis, particularmente nas redes familiares de doação, nas quais candidatos aparentados podem compartilhar hábitos familiares, ambientais e socioeconômicos, além de fatores comportamentais relacionados ao risco renal e cardiometabólico^15,16,17,18^.

Além disso, a avaliação contemporânea do doador vivo vai além da exclusão de doenças clinicamente manifestas e também incorpora a estimativa de risco futuro. Assim, fatores de risco previamente não diagnosticados, subestimados ou insuficientemente monitorados podem emergir apenas durante o processo de avaliação pré-transplante, tornando a seleção do doador mais prolongada, mais intensiva em recursos e emocionalmente onerosa, especialmente quando o candidato, ao final, não é aprovado para a doação. No entanto, essa interpretação deve ser compreendida como uma hipótese contextual plausível, e não como evidência explicativa direta.

As comparações internacionais são informativas a esse respeito. Turquia, Israel e República da Coreia são três dos quatro países mais bem classificados em transplante renal com doador vivo, e ilustram como contextos culturais, econômicos, legais e organizacionais podem estar associados a diferentes padrões de doação intervivos^14^.

Na Turquia, a predominância da doação intervivos é interpretada no contexto de proteção ao doador e da organização do sistema de saúde local, incluindo regulamentações trabalhistas que podem contribuir para a preservação do vínculo empregatício durante a recuperação do doador, benefícios por afastamento por doença nesse período e cobertura pública da avaliação do doador, da cirurgia, da hospitalização e do seguimento pós-doação, no âmbito de um sistema nacional de transplantes estruturado^19^.

Em Israel, a legislação sobre transplantes e os mecanismos de apoio ao doador incluem, segundo relatos, compensação de custos, reembolso de perdas salariais, isenção temporária de taxas relacionadas à saúde, cobertura estatal de seguros de vida e invalidez, além de prioridade futura na lista de espera para transplante. Além disso, Israel apresenta elevada proporção de doações renais intervivos provenientes de doadores não parentes, característica associada, na literatura, à cultura altruísta de doação e à existência de mecanismos eficazes de apoio aos doadores vivos^20^.

Na República da Coreia, a maioria dos doadores renais vivos é composta por familiares, e o transplante renal com doador vivo é relacionado a fatores culturais e demográficos, incluindo percepções influenciadas pelo confucionismo sobre a integridade corporal após a morte, valores centrados na família, como responsabilidade e obrigação mútua, mudanças demográficas, redução das taxas de algumas das principais causas de morte encefálica e envelhecimento populacional^21^.

Esses exemplos são pertinentes ao Brasil, uma vez que algumas barreiras podem persistir mesmo quando a assistência médica direta é coberta pelo sistema público. Embora o SUS cubra os exames do doador, a cirurgia, a hospitalização e o seguimento pós-doação, permanecem barreiras indiretas, incluindo a ausência de proteção específica à estabilidade no emprego para doadores vivos e mecanismos de afastamento médico que podem beneficiar os trabalhadores com vínculo formal de emprego.

Os trabalhadores informais, por sua vez, podem permanecer particularmente vulneráveis. Dados da PNAD Contínua de 2023, último ano do período deste estudo, registraram 39,4 milhões de trabalhadores informais e uma taxa de informalidade de 39,2%^22^. Assim, o afastamento do trabalho, os custos com transporte, as responsabilidades de cuidado e a insegurança de renda podem representar barreiras socioeconômicas e logísticas para potenciais doadores.

Paralelamente, a série brasileira também evidenciou mudanças no perfil de vínculo doador–receptor entre os transplantes renais com DV. A categoria *Parente* permaneceu como a principal fonte de doação intervivos ao longo de toda a série; entretanto, sua participação proporcional diminuiu de 82,80% em 2010 para 71,21% em 2023. Em contraste, a participação proporcional da categoria *Não Parente: Cônjuge* aumentou de 10,57% para 18,65%, e a de *Não Parente: Outros* aumentou de 6,64% para 10,14%, embora os números absolutos tenham diminuído em ambas as categorias *Não Parente*.

Esse ponto é relevante para os presentes resultados porque o declínio do transplante renal com DV se concentrou nas categorias *Parente* e *Não Parente: Cônjuge*, grupos para os quais não é exigida autorização judicial^23^; em conjunto, essas categorias responderam por 97,1% da redução total observada no período estudado. Esse resultado sugere que estratégias educacionais, organizacionais e de proteção ao doador devem ser consideradas em paralelo ao debate regulatório.

A mudança na composição da doação intervivos é clinicamente relevante porque, na presente análise descritiva, as estimativas de sobrevida publicadas pelo RBT/ABTO para o mesmo período analisado neste estudo, 2010 a 2023, mostraram que, em até 14 anos de seguimento, receptores de DV apresentaram sobrevida do paciente e do enxerto superiores às dos receptores de DF em 16 e 21 pontos percentuais, respectivamente^13^. Contudo, essas estimativas baseadas em registro são descritivas e podem refletir diferenças na seleção de doadores e receptores, nos perfis clínicos, no tempo em diálise, na compatibilidade imunológica, no tempo de isquemia e na logística do procedimento.

Os indicadores hospitalares também mostraram um perfil descritivo favorável ao transplante com DV. Na base SIH/SUS^11^, as internações associadas ao DV apresentaram menor tempo de permanência hospitalar (−19,51%), menor mortalidade intra-hospitalar (−73,53%) e menores valores médios de reembolso por AIH (−31,46%) em comparação às internações associadas ao DF, em todas as macrorregiões. Esse perfil pode refletir a previsibilidade logística e o caráter eletivo de muitos procedimentos com DV, sem implicar causalidade; os valores de AIH correspondem a montantes nominais registrados e reembolsados e podem não captar os custos econômicos totais, particularmente aqueles relacionados à avaliação do doador, à nefrectomia, ao seguimento pós-doação e a eventuais intercorrências.

Embora descritivos, esses indicadores clínicos e hospitalares sugerem a relevância de identificar barreiras ao transplante com DV que possam ser abordadas por meio de intervenções no sistema de saúde. Nesse sentido, intervenções educacionais representam uma estratégia para enfrentar barreiras potencialmente modificáveis ao transplante com DV^24,25^. No estudo *Renal Education and Choices at Home* (ReACH)^25^ *,* um programa domiciliar conduzido por enfermeiros especialistas, direcionado a pacientes, familiares e amigos, teve como objetivo ampliar o conhecimento, promover o diálogo sobre doação e reduzir barreiras ao transplante. Após a intervenção, 53% dos pacientes passaram a ter um ou mais candidatos a doador em processo de avaliação.

De forma consistente com esses achados, em uma revisão qualitativa de 27 estudos internacionais, Truhan et al.^26^ identificaram a oferta tardia ou fragmentada de informações sobre a doação intervivos como uma barreira recorrente, associada à incerteza, à superestimação dos riscos para o doador e a dificuldades na comunicação familiar. Os autores enfatizaram a importância de uma educação precoce, contínua, centrada na família e mediada por profissionais, com envolvimento da rede familiar desde as etapas iniciais^26,27^.

Além das estratégias educacionais, a doação renal pareada tem sido proposta como uma alternativa para ampliar as oportunidades de transplante com DV em sistemas altamente estruturados. No entanto, Medina-Pestana et al.^28^ descrevem barreiras à sua ampla implementação nos contextos latino-americano e brasileiro, incluindo desigualdades socioeconômicas, assimetrias de acesso e desafios éticos, logísticos e de equidade. Ainda assim, iniciativas recentes, como o Projeto de Lei nº 3.903/2024, indicam que o debate regulatório nacional sobre a doação pareada permanece em curso^29^.

Com base nos achados descritivos apresentados, é razoável considerar ações integradas de natureza educacional, organizacional e regulatória para apoiar a doação renal intervivos e aprimorar as vias de acesso ao transplante, em paralelo ao fortalecimento contínuo da captação de órgãos de doadores falecidos e de estratégias voltadas à redução da pressão sobre a lista de espera para transplante renal, que constitui a maior lista de espera por órgão específico no Brasil. Nesse contexto, iniciativas de conscientização e medidas de apoio ao doador eticamente apropriadas devem ser consideradas nos processos decisórios do SUS e das operadoras de planos de saúde.

### Limitações do estudo

Este estudo apresenta limitações inerentes ao uso de dados secundários, agregados e administrativos, que restringem as análises ao nível populacional e impedem a avaliação de determinantes individuais da doação intervivos, como idade, sexo, escolaridade, vínculo familiar e condições clínicas. O uso de diferentes sistemas de informação, SNT, RBT/ABTO e SIH/SUS, pode resultar em divergências nas definições operacionais, nos procedimentos de fechamento anual, na completude e na consistência dos registros, com potencial para subnotificação, variabilidade entre regiões e instituições e viés de informação. As medidas da lista de espera do SNT descrevem o estoque no Registro Técnico e não capturam a dinâmica de entradas e saídas ao longo do tempo. Além disso, as comparações entre modalidades de doação são descritivas e não ajustadas para diferenças no perfil clínico, na complexidade assistencial, no caráter eletivo dos procedimentos e em fatores logísticos. Por fim, os indicadores do SIH/SUS refletem internações realizadas no âmbito do SUS, com mortalidade restrita ao período de hospitalização, e o valor médio da AIH representa o montante registrado e reembolsado em termos nominais e não o custo econômico total. Mudanças administrativas, de codificação e de reembolso ao longo do período podem afetar a comparabilidade dos resultados.

## CONCLUSÃO

Entre 2010 e 2023, o transplante renal no Brasil apresentou uma mudança expressiva no perfil de doadores, caracterizada pelo aumento dos procedimentos com doador falecido e pela redução dos procedimentos com doador vivo. No mesmo período, o estoque da lista de espera para transplante renal permaneceu substancialmente superior à produção anual de transplantes, sugerindo uma carga operacional persistente sobre o sistema de transplantes. Entre os transplantes com doador vivo, os dados também indicaram mudanças nas categorias de vínculo doador–receptor, com declínio mais acentuado na categoria Parente e aumento da participação relativa dos doadores Não Parente, particularmente da categoria Não Parente: Cônjuge. Os indicadores clínicos e hospitalares foram descritivamente mais favoráveis entre receptores de transplante renal com doador vivo, incluindo maior sobrevida do paciente e do enxerto, menor tempo de permanência hospitalar, menor mortalidade intra-hospitalar e menores valores médios de AIH. Em conjunto, esses achados sugerem a relevância de estratégias integradas para fortalecer o transplante renal com doador vivo, em paralelo à continuidade dos esforços para sustentar a captação de órgãos de doadores falecidos. Contudo, este estudo é descritivo e não deve ser interpretado como evidência causal.

## Data Availability

Os conjuntos de dados analisados no presente estudo foram derivados de fontes publicamente disponíveis, citadas no manuscrito.
